# Healthcare Workers Occupationally Exposed to Ionizing Radiation Exhibit Altered Levels of Inflammatory Cytokines and Redox Parameters

**DOI:** 10.3390/antiox8010012

**Published:** 2019-01-01

**Authors:** Iman M. Ahmad, Maher Y. Abdalla, Tiffany A. Moore, Lisa Bartenhagen, Adam J. Case, Matthew C. Zimmerman

**Affiliations:** 1 Department of Medical Imaging and Therapeutic Sciences, College of Allied Health Professions, University of Nebraska Medical Center (UNMC), Omaha, NE 68198, USA; labarten@unmc.edu; 2 Department of Pathology and Microbiology, College of Medicine, UNMC, Omaha, NE 68198, USA; maher.abdalla@unmc.edu; 3 College of Nursing, UNMC, Omaha, NE 68198, USA; tamoore@unmc.edu; 4 Department of Cellular and Integrative Physiology, College of Medicine, UNMC, Omaha, NE 68198, USA; adam.case@unmc.edu (A.J.C.); mczimmerman@unmc.edu (M.C.Z.)

**Keywords:** O_2_•^−^, EcSOD, inflammatory cytokines, oxidative stress, DNA oxidation, glutathione

## Abstract

Studies have shown an increased risk for a variety of cancers, specifically brain cancer, in healthcare workers occupationally exposed to ionizing radiation. Although the mechanisms mediating these phenomena are not fully understood, ionizing radiation-mediated elevated levels of reactive oxygen species (ROS), oxidative DNA damage, and immune modulation are likely involved. A group of 20 radiation exposed workers and 40 sex- and age-matched non-exposed control subjects were recruited for the study. We measured superoxide (O_2_•^−^) levels in whole blood of healthcare workers and all other measurements of cytokines, oxidative DNA damage, extracellular superoxide dismutase (EcSOD) activity and reduced/oxidized glutathione ratio (GSH/GSSG) in plasma. Levels of O_2_•^−^ were significantly higher in radiation exposed workers compared to control. Similarly, a significant increase in the levels of interleukin (IL)-6, IL-1α and macrophage inflammatory protein (MIP)-1α in radiation exposed workers compared to control was observed, while there was no significance difference in the other 27 screened cytokines. A significant positive correlation was found between MIP-1α and O_2_•^−^ levels with no correlation in either IL-6 or IL-1α. Further, a dose-dependent relationship with significant O_2_•^−^ production and immune alterations in radiation exposed workers was demonstrated. There was no statistical difference between the groups in terms of oxidative DNA damage, GSH/GSSG levels, or EcSOD activity. Although the biologic significance of cytokines alterations in radiation exposed workers is unclear, further studies are needed for determining the underlying mechanism of their elevation.

## 1. Introduction

Ionizing radiation is used commonly in medical diagnostics, and the advancement of diagnostic imaging and interventional radiology has raised concern about the potential risk these advancements may pose to healthcare workers utilizing these technologies. Today, surveillance of healthcare workers chronically exposed to ionizing radiation only provides information on accidental overexposure, not on the real chronic risk of exposure to low dose ionizing radiation. Therefore, there is an urgent need to closely examine potential pathological changes occurring in workers chronically exposed to ionizing radiation. Although occupational exposure to ionizing radiation generally falls well below the currently accepted limits (i.e., less than 50 millisieverts (mSV)) set by the International Commission of Radiation Protection (ICRP) [1], several epidemiological studies of radiation exposed workers have indicated an increased risk for a variety of cancers, specifically, more than two-fold for brain cancer [2]. The mechanisms mediating these phenomena are complex and likely involve elevated levels of reactive oxygen species (ROS), oxidative DNA damage, and immunosuppression triggered by ionizing radiation [3,4,5,6].

We previously reported that occupational exposure to ionizing radiation even within the limits of ICRP recommendations results in an alteration of redox environment with an increase in ROS, particularly superoxide (O_2_•^−^) [7]. Further, it has been shown that chronic oxidative stress contributes to many pathological conditions including inflammation, fibrosis, and necrosis [8], as well as DNA damage and cancer [9,10,11]. In addition, the potential mutagenic and carcinogenic risk of ionizing radiation exposure has been documented [3,4] and discussed in [12]. Strong evidence indicates that radiation induces carcinogenesis, predominantly by causing DNA damage, thereby leading to chromosome instability and carcinogenesis [3,4,12]. It must not go unnoticed that there are other factors contributing to radiation-induced carcinogenesis such as non targeted effects, inflammation, as well as constant activation of the immune system as reviewed in [12].

A growing body of evidence indicates immunological changes induced by exposure to ionizing radiation. The effect of ionizing radiation on selected indices of cellular and humoral immunities in workers occupationally exposed to low levels of ionizing radiation has been studied [13,14]. Cluster of differentiation (CD)4(+) T-lymphocytes and humoral immune response levels were found to be significantly lower in radiation exposed workers compared to control group [13]. Further, studies on the effect of low doses of ionizing radiation exposure on peripheral blood lymphocytes has shown a significant increase of serum interleukin (IL)-2 and decrease of serum IL-4 in radiation exposed workers compared to controls [13]. Other studies performed in mice, indicate that lymphocytes are vulnerable to acute and chronic radiation exposure, and immunosuppression is triggered by chronic exposure to ionizing radiation [15]. Therefore, it is important to periodically check immune response levels in radiation exposed workers to detect any early immune deficiencies. However, available studies do not reflect how these effects change with different occupational settings having different radiation doses in radiography. Furthermore, no data exists towards elucidating the relationship between immune response alterations and O_2_•^−^ levels in radiation exposed workers. In view of the above considerations, the present study was designed to assess the plasma inflammatory cytokines level, oxidative DNA damage, and antioxidants to determine their association with O_2_•^−^ levels in radiation exposed workers at different occupational settings.

## 2. Materials and Methods

### 2.1. Samples Collection

Blood samples were collected from 60 healthy workers: 20 of them occupationally exposed to radiation (all radiologic technologists, age = 39.4 ± 2.19 years, healthcare employees of several sectors Conventional Radiography (CR, *n* = 12), Interventional Radiography (IR, *n* = 4), and Computed Tomography (CT, *n* = 4), while the remaining 40 samples were from unexposed individuals’ age- and gender matched (Table 1). All subjects completed a detailed questionnaire that included personal information (age, medication, and health status), lifestyle (smoking, alcohol consumption, exercise), and X-ray exposure as a patient. Total lifetime radiation effective dose over years of occupational exposure was obtained and calculated following recommendations of the National Council of Radiation Protection [16] (expressed in mSV). The study was approved by the Institutional Review Board of University of Nebraska Medical Center (Protocol No. 222-14-EP), and informed consent was obtained.

### 2.2. Blood Collection

Blood was collected from all participants into ethylenediaminetetraacetic acid (EDTA) tubes. Blood plasma and red blood cells were separated by centrifugation of the whole blood at 2500× *g* at 4 °C for 5 minutes, washed and then stored at −80 °C until analyzed.

### 2.3. Superoxide Measurement

Total cellular O_2_•^−^ levels were assessed as described in our earlier study [7]. 100 μL of whole blood immediately after sample collection, was incubated with a superoxide- sensitive electron paramagnetic resonance (EPR) spin probe, 1-hydroxy-3-methoxycarbonyl-2,2,5,5-tetramethylpyrrolidine (CMH) (200 μM; 60 min; 37 °C then frozen in liquid nitrogen) dissolved in EPR buffer (Krebs Hepes Buffer (KHB)), supplemented with metal chelators sodium diethyldithiocarbamate trihydrate (DETC, 5 μM) and deferoxamine (DF, 25 μM pH 7.4). EPR measurements were performed with a Bruker eScan EPR spectrometer (Bruker BioSpin GmbH, Rheinstetten/Karlsruhe, Germany), with the following parameters: field sweep width, 100.0 G; center field, 3482 G; microwave frequency, 9.75 kHz; microwave power, 1.10 mW; modulation amplitude, 5.94 G; conversion time, 10.24 ms; time constant, 40.96 ms. The EPR spectrum amplitude intensity was defined as peak-to-peak height and expressed as arbitrary units (a.u.).

### 2.4. Assessment of DNA Oxidation

DNA oxidation (8-hydroxydeoxyguanosine (8-OHdG)) was assessed in blood plasma using commercially available assay, OxiSelect^TM^ Oxidative DNA Damage ELISA Kit (8-OHdG Quantitation, Cell Biolabs, Inc., San Diego, CA, USA), and per the manufacturer’s instructions. Concentrations were expressed as ng/mL. The assay detection sensitivity ranges from 100 pg/mL to 20 ng/mL.

### 2.5. Cytokine Levels

Electrochemiluminescence-based immunoassay was used to measure cytokine levels in plasma. Samples and standards were prepared on multispot 96-well plates from the V-PLEX^®^ Human Cytokine 30-Plex Kit (Meso Scale Discovery^®^, Rockville, MD, USA) per the manufacturer’s instructions. Plates were then analyzed by the Meso Scale Discovery® (MSD) QuickPlex SQ 120 and samples concentrations were calculated using the Discovery Workbench 4.0 using a 4-PL curve fit model. Samples below the lower level of detection, which was calculated by the workbench software as 2.5 standard deviations above the assay background blank, were reported as 0 pg/mL.

### 2.6. Extracellular Superoxide Dismutase (EcSOD) and Glutathione Levels

Reduced glutathione (GSH) and oxidized glutathione (GSSG) Levels in blood plasma were measured using a commercially available assay (GSSG/GSH Quantification kit; Dojindo, Inc. Rockville, MD, USA). Activity of EcSOD in plasma was measured using a superoxide dismutase (SOD) Assay Kit from Dojindo (Inc. Rockville, MD, USA), according to the manufacturers’ guidelines.

### 2.7. Statistical Analysis

GraphPad Prism version 5 (GraphPad Software, San Diego, CA, USA) was used for statistical analysis. Data are presented as mean ± standard error of the mean (SEM). Comparison between two groups was performed with the Mann- Whitney test. Comparison between three or more groups was done by One-way analysis of variance with Bonferroni post hoc tests. Pearson’s correlation test was performed to identify the relationships between variables. A *p* value < 0.05 was considered significant for all statistical analyses.

## 3. Results

### 3.1. Study Participants

Demographic characteristics of the study subjects are not statistically different between control and radiation exposed workers as shown in Table 1. The mean age of the control and radiation exposed workers was 41.1 ± 1.8 and 39.4 ± 2.2 years, respectively, with no significant differences (Table 1). The average work experience of radiation exposed group was 16 ± 2 years. Alcohol intake, dietary supplements and exercise level did not differ significantly between the two groups. All participants are non-smokers. The average annual dose levels of radiation exposed workers, 2.03 mSv, are below the limits set by the International Commission of Radiation Protection (ICRP) [1]. Radiation exposed workers were further subdivided into three groups with their mean lifetime effective radiation doses (mSv) calculated from their personal dosimetry: CR (17.09 ± 5.73), IR (31.00 ± 16.17), and CT (45.98 ± 11.32).

### 3.2. Superoxide (O_2_•^−^) Level

**Figure 1:**
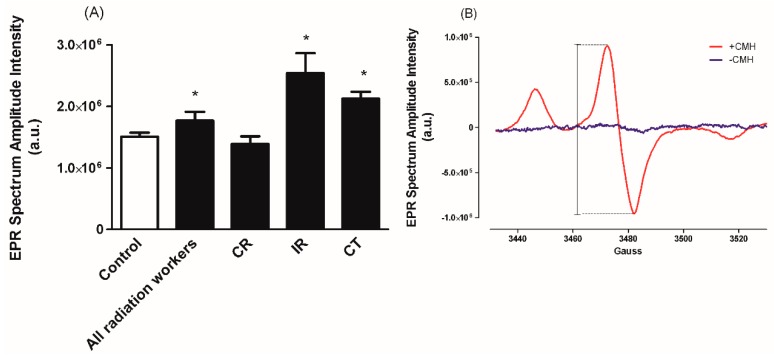
Summary data showing O_2_•^−^ levels, reported as electron paramagnetic resonance (EPR) Spectrum Amplitude Intensity, in whole blood of subjects exposed to occupational ionizing radiation (**A**), and representative EPR spectrum from whole blood samples with (red spectrum) or without (blue spectrum) the 1-hydroxy-3-methoxycarbonyl-2,2,5,5-tetramethylpyrrolidine (CMH) spin probe (**B**). The EPR spectrum amplitude is directly proportional to the levels of O_2_•^−^ in the sample and was quantified as peak-to-peak amplitude intensity as indicated in (**B**). CR: conventional radiography, IR: interventional radiography, CT: computed tomography. a.u. = arbitrary unit. Data represent the mean ± standard error of the mean (SEM). * *p* < 0.05 versus control and CR [modified from [7]].

As we previously reported [7], O_2_•^−^ level in whole blood of radiation exposed workers was significantly higher compared with the control subjects, Figure 1A. With respect to the occupational setting subgroups, a marked difference was seen between IR and CT subgroups compared to control and CR subgroups (*p* < 0.05, Figure 1A). However, insignificant difference was seen between CR subgroup compared with control subjects and IR compared with CT (*p* > 0.05, Figure 1A). As shown in the representative EPR spectrum with and without the CMH spin probe (Figure 1B), we did not detect ascorbyl radical or any other radicals in the control samples (i.e., no CMH).

### 3.3. Systemic Inflamzmatory Marker Analysis

**Figure 2:**
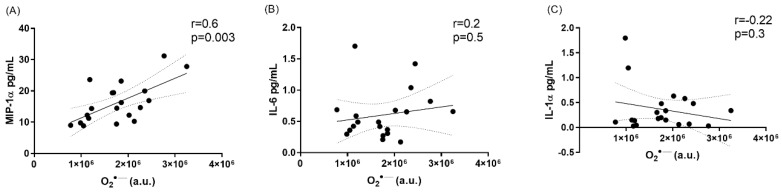
Association between MIP-1α, IL-6 and IL1-1α and O_2_•^−^ in radiation exposed workers. (**A**), Correlation analysis of MIP-1α and O_2_•^−^ (*r* = 0.6). (**B**), Correlation analysis of IL-6 and O_2_•^−^, (*r* = 0.2). (**C**), Correlation analysis of IL-1α and O_2_•^−^, (*r* = −0.22).

A list of all cytokines with corresponding mean values in the plasma are listed in Table 2. Of the thirty cytokines measured, only three (IL-6, macrophage inflammatory protein (MIP)-1α and IL-1α) were signficantly (*p* < 0.05) different betweeen radiation exposed workers and control subjects (Table 2). A significant increase of IL-6 and MIP-1α was found in the IR subgroup compared to control and CR subgroups (*p* < 0.05, Table 3). Although there was no significant difference between the CT subgroup versus control and CR subgroups, there is a trend of increase. Further, there was no significant difference between IR and CT subgroups or control subjects and CR subgroup (*p* > 0.05, Table 3). In addition, of the three cytokines mentioned above, MIP-1α correlated positively with O_2_•^−^ levels (*r* = 0.6, *p* < 0.003), whereas both IL-6 and IL-1α did not (*r* = 0.2, *r* = −0.22, *p* > 0.05) (Figure 2).

### 3.4. Plasma 8-OHdG Concentration

**Figure 3:**
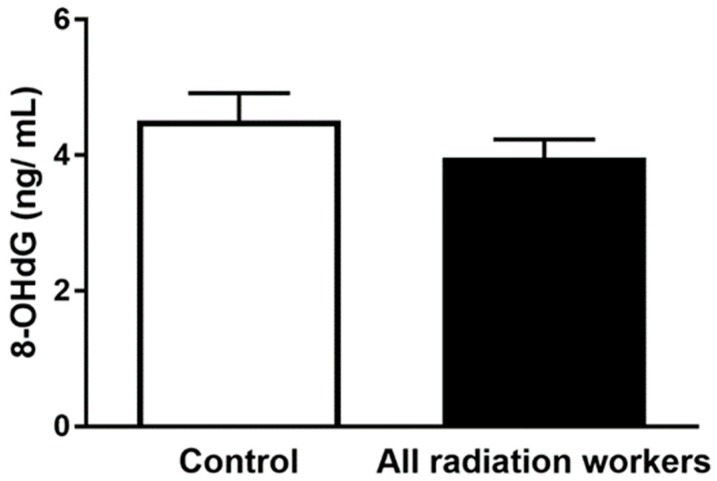
Mean values of plasma 8-OHdG concentration (ng/ mL) in all radiation workers compared to control group. Data represent the mean ± SEM, *p* > 0.05.

The results of plasma 8-OHdG in radiation workers subgroups compared to control group are illustrated in Table 4 and Figure 3. Analysis of 8-OHdG levels within control subjects and all radiation exposed workers revealed no significant differences (*p* > 0.05). Moreover, there were insignificant differences in plasma 8-OHdG between CR, IR and CT subgroups, (*p* > 0.05). There was no correlation between 8-OHdG and O_2_•^−^ levels.

### 3.5. Antioxidants Levels

**Figure 4:**
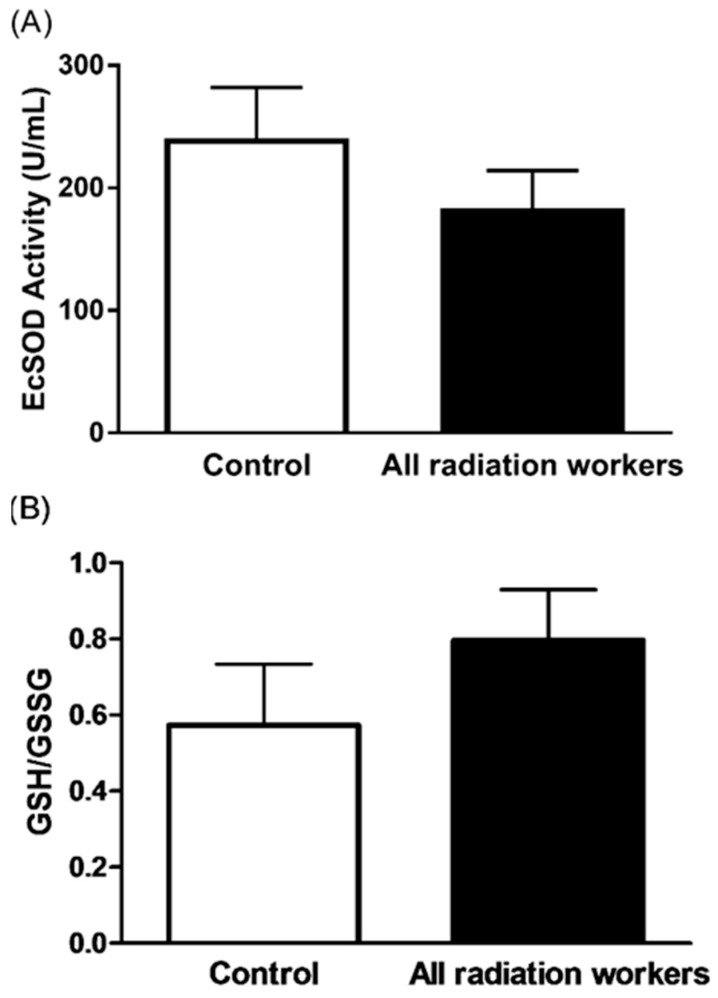
Mean values of plasma EcSOD activity (**A**) and the GSH/GSSG ratio (**B**) from all radiation workers and control subjects. Data represent the mean ± SEM, *p* > 0.05.

Owing to elevated levels of O_2_•^−^ in radiation exposed workers, it would be expected to observe a difference in antioxidant levels in radiation workers compared to control group. We have previously [7] reported that workers exposed to radiation have higher intracellular blood levels of SOD, however, in our current study, the level of EcSOD activity in the radiation exposed and control group was 183.1 ± 31.1 and 240.4 ± 41.5 U/mL, respectively, and the difference was not statistically significant (*p* > 0.05, Figure 4A). These results suggest an intracellular protective mechanism to compensate for elevated levels of O_2_•^−^. Similarly, no significant difference was observed between the two study groups in GSH/GSSG ratio (*p* > 0.05, Figure 4B). There was a significant negative correlation between EcSOD activity and O_2_•^−^ level (*r* = −0.5, *p* = 0.04). However, the association of GSH/GSSG with O_2_•^−^ level failed to demonstrate any association (*r* = −0.03, *p* = 0.8).

## 4. Discussion

The effect of occupational exposure to low levels of ionizing radiation is a serious concern to a large number of radiation workers [17]. Although radiation exposed workers are not directly exposed to radiation, they receive scatter radiation that is extremely variable [18,19,20]. While, occupational exposure to ionizing radiation has remained within the currently accepted limits set by ICRP [1], an increased risk of leukemia and multiple myeloma or solid cancers, has been reported [2,21,22]. The mechanisms mediating these phenomena are complex and likely involve elevated levels of ROS, oxidative stress, DNA damage, and immunosuppression triggered by ionizing radiation [3,4,5,6]. The biological effects of ionizing radiation are induced either directly by damaging the DNA or indirectly by generating ROS that count for the 70% of all biological effects [23,24]. In this study, we assessed ROS in whole blood from radiation exposed workers by measuring O_2_•^−^ levels using EPR spectroscopy. Our data reveal that in IR and CT subgroups (high dose radiologic procedures) compared to CR subgroup (low dose radiologic procedures), chronic exposure to ionizing radiation is associated with an increase in O_2_•^−^. Considering we used whole blood for these measurements, it is likely that lymphocytes and/or erythrocytes are the source of increased levels of O_2_•^−^.

Our body has a variety of defense mechanisms comprising antioxidant enzymes to counteract ROS-mediated oxidative damage [25,26]. Therefore, periodic checks of oxidative stress, DNA damage and immune response levels, thought to play a central role in development of cancer, in radiation exposed workers could be of importance to guide health promotion and disease prevention. In the present study, we investigated the effect of occupational radiation exposure on immune response alterations, DNA oxidation and extracellular antioxidants level in radiation exposed workers at different occupational settings. We demonstrate that levels of IL-6, MIP-1α, and IL-1α are remarkably increased in radiation exposed workers compared to the control subjects. Yet, we did not find significant differences in other cytokines between control subjects and radiation exposed workers. A significant positive correlation was found between MIP-1α and O_2_•^−^ levels with no correlation in either IL-6 or IL-1α. Our current understanding of the importance of immune system’s role in cancer control, studies of radiation-immune system interactions have been one of the main research fields in radiation biology and radiation protection. However, there has been little research examining the dose–response relationship of ionizing radiation-induced immune alterations. Interestingly, our data has showed a significant increase in MIP-1α in the plasma of radiation exposed workers especially in IR subgroup (high-dose radiologic procedures), compared to CR subgroup (low dose radiologic procedures) and control, which suggests an association between inflammation and high radiation absorbed dose in the radiation exposed workers. Acute inflammation is the initial protective response by the body; however, chronic inflammation can lead to pathology. Proper regulation of cytokine production is critical in diseases control and prevention [27]. In our study, although the annual exposure doses were within limits recommended by ICRP, we found a dose-dependent relationship with significant immune changes. Our results support the existence of a threshold dose at 17 mSv for radiation-related health effect as shown by no differences in the levels of MIP-1α between the CR and control groups. Exposure above the threshold dose showed an increased response, and linearity disappears at doses higher than 31 mSv as shown by no changes in the levels of MIP-1α between the IR and CT subgroups. This experimental data is inconsistence with the linear-non threshold (LNT) model [28] as discussed in our previous work showing similar trend with O_2_•^−^ levels [7] and should be interpreted with caution. Additionally, these radiation-induced alterations in radiation exposed workers demonstrate a critical need for defining the safest radiation dose with no observed biological effects. Our results suggest pro-inflammatory response and our findings are in agreement, to some extent, with previous studies. Zakeri et al., showed a significant serum increase of IL-2 and decrease of IL-10 in the Interventional cardiologists group compared with the control group [29]. Hrycek et al., showed significantly higher serum levels of IL-2 and lower levels of IL-4 in radiation workers compared to the control group [14]. In addition, mice studies showed that low doses of ionizing radiation increased IL-12 and decreased IL-10 secretions [30]. Another study in mice has also demonstrated an in vivo inflammatory cytokines production in response to activation of resident peritoneal macrophages following exposure to low dose γ-irradiation [31]. Shieh et al., showed also an increased IL-2 secretion in mice exposed to a single low dose ionizing radiation [32]. Although the mice model of a single low dose ionizing radiation exposure, is different from the long term low dose ionizing radiation effects, these studies indicate a positive biological effect on the immune system and might improve understanding the mechanisms underlying these effects.

With respect to DNA oxidation, the current study showed that the mean values of plasma 8-OHdG in all radiation exposed workers compared to control group were insignificant, with no significant values observed among IR compared to CR or CT (Table 4). This finding is contrary to our hypothesis that exposure to chronic low dose radiation induces oxidative stress increasing vulnerability to DNA oxidative damage. 8-OHdG is one of the predominant forms of free radical-induced oxidative damage and has been used to estimate oxidative stress-related DNA damage in humans after ionizing radiation exposure. However, plasma 8-OHdG levels could also be influenced by the rate of repair and not only by the rate of damage and ultimately we need to use other techniques in the future to assess DNA damage at nuclear and cellular levels. Previous studies revealed an increase of the concentration of 8-OHdG in urine of radiation exposed workers compared to controls [33]. El-Benhawy et al., found that, serum 8-OHdG in radiation exposed workers was significantly higher compared to control group [34]. These discrepancies might be due, in part, to the source and dose of radiation, as well as, to different target groups’ studies and sample collection.

In general, the link between radiation-induced DNA damage and immune response has been demonstrated in many cases as reviewed in [35,36]. Previously, it has been shown that cellular DNA damage can release several cytokines involved in regulation of immune responses such as IL-6 [12,37]. According to our results, we believe that low dose radiation exposure can induce cellular damage –probably by inducing ROS- and this, in turn, induced cytokine production through innate- adaptive immune response. As discussed before, further studies still needed to identify intra-cellular DNA damage markers and other markers of activated immune response.

Earlier, we demonstrated that occupational radiation exposure within ICRP recommendation limits [1], results in redox balance alterations as evidenced by significant increase in O_2_•^−^ and lipid peroxidation [7]. These findings of oxidants increase are accompanied by an increase in intracellular SOD activity. These observations were more pronounced in CT and IR subgroups (high dose radiologic procedures) compared to CR subgroup (low dose radiologic procedures) [7]. The current study continued this line of investigation by looking at the extracellular level of antioxidants (EcSOD, GSH/GSSG). Our bodies are well equipped with antioxidants to combat the oxidative stress challenge [38]. GSH is the most abundant antioxidant in our body. The ratio of reduced to oxidized glutathione (GSH/GSSG) has often been used as markers of oxidative stress, and alterations in this ratio have been shown in various diseases including aging, cancer, human immunodeficiency virus (HIV) replication [39,40,41,42,43], cardiovascular diseases and in neurodegenerative diseases, such as Parkinson disease and Alzheimer disease [44]. Therefore, measuring GSH/GSSG ratio is the best assessment for any cellular redox alterations. Our study demonstrates no changes in the ratio of GSH/GSSG between radiation exposed workers and control subjects. Although these data are in accordance with others [45,46], it should be noted that other previous studies have reported increased GSH levels in the blood of radiation exposed workers [47]. Superoxide dismutase is an important antioxidant enzyme that catalyzes the dismutation of O_2_•^−^ into H_2_O_2_ to reduce ROS-mediated diseases such as carcinoma, inflammation, and aging [48,49]. Previous studies have observed the increased SOD activity in the blood plasma of radiation exposed workers [45,46]. Our findings are in disagreement with these studies, where radiation exposed workers have shown no changes in EcSOD activity. Similar to the discrepancy in the literature regarding changes in 8- OHdG levels, these differences in EcSOD activity and GSH/GSSG may be due to differences in work-related tasks assigned to radiation exposed workers as well as differences in the radiation doses these workers are exposed to.

It must be noted there are limitations in our study. A larger sample size would allow more statistical significance in the evaluation of biomarkers. Also, using 8-OHdG to estimate DNA damage in humans after ionizing radiation exposure is another limitation in our study as plasma 8-OHdG levels could be influenced by the rate of repair as well as by the rate of damage and ultimately we need to use other techniques in the future to assess DNA damage at nuclear and cellular levels. Further, CMH is not 100% specific for O_2_•^−^, as there is evidence that it reacts with peroxynitrite, nitrogen dioxide, and peroxyl radical. However, it should be noted that O_2_•^−^ has the highest interaction constant with CMH [50,51]. As such, we speculate we have detected O_2_•^−^ with CMH and this supports our conclusion that there is increased oxidative stress in our studied subjects. Lastly, we posit that the increase in O_2_•^−^ levels observed in whole blood is likely from lymphocytes; however, without knowing the exact white blood cells profile in our samples, it is difficult to make strong conclusions regarding the precise source of O_2_•^−^.

## 5. Conclusions

In conclusion, the results presented in this study have demonstrated a dose-dependent relationship with significant O_2_•^−^ production and immune alterations in radiation exposed workers and more specifically in high dose radiologic procedures (i.e., IR). Although the biologic significance of changes in these cytokines is unclear, results from the current study indicate the importance to take all necessary measures to protect radiation exposed workers from radiation exposure. The underlying mechanism of their elevation needs further investigation. In view of the importance to improve understanding of the long-term health effects in workers occupationally exposed to radiation, Low dose radiation effect studies have to be one of the main research priority. Thus, follow-up evaluation of occupational health status, should be considered an integral part of quality assurance programs.

## Figures and Tables

**Table 1:** Characteristics of the study subjects included in the study [7].

Characteristics	Control (*n* = 40)	All radiation workers (*n* = 20)	*p*-Value
Age (Mean ± standard error of the mean (SEM))	41.1 ± 1.8	39.4 ± 2.2	0.57
Gender			1
Male (%)	5 (12.5)	3 (15)	
Female (%)	35 (87.5)	17 (85)	
Alcohol intake (%)			0.4
Yes	28 (70)	17 (85)	
No	12 (30)	3 (15)	
Smoking			
Yes	0	0	
No	40 (100%)	20 (100%)	
Dietary Supplements (%)			0.2
Yes	18 (45)	16 (80)	
No	22 (55)	4 (20)	
Mean dose (millisieverts (mSv)/year)—(SEM)	0	2.03 (0.4)	
Duration of radiation exposure, years (Mean ± SEM)	NA	16 ± 2	
GLTEQ total-mean (SEM)	35.6 (3.3)	38 (6.4)	0.7
GLTEQ sweat/heart beat			0.15
(i) Never	10	1	
(ii) Sometimes	20	14	
(iii) Often	10	5	

GLTEQ: Godin-Leisure-Time Exercise Questionnaire.

**Table 2:** Plasma cytokines level in control subjects and all radiation workers (pg/mL).

Cytokines	Control	All Radiation Workers	*p*-Value
IFN-γ	10.2 ± 1.84	6.56 ± 0.84	0.24
IL-10	0.51 ± 0.18	0.38 ± 0.07	0.67
IL-12p70	0.15 ± 0.05	0.12 ± 0.03	0.64
IL-13	0.09 ± 0.05	0.1 ± 0.04	0.61
IL-1β	0.14 ± 0.13	0.01 ± 0.01	0.94
IL-2	0.12 ± 0.02	0.30 ± 0.21	0.91
IL-4	0.12 ± 0.08	0.01 ± 0.00	0.44
IL-6	0.44 ± 0.08	0.60 ± 0.08 *	0.04
IL-8	5.28 ± 0.49	4.85 ± 0.54	0.62
TNF-α	2.45 ± 0.31	2.16 ± 0.16	0.64
Eotaxin	742 ± 60.1	711 ± 49.1	0.70
Eotaxin-3	105 ± 7.45	110 ± 9.88	0.74
IL-8 (HA)	27.4 ± 15.6	13.7 ± 4.78	0.96
IP-10	478 ± 210	273 ± 35.7	0.55
MCP-1	103 ± 9.51	97.8 ± 5.06	0.76
MDC	613 ± 33.4	599 ± 58.8	0.43
MIP-1α	12.0 ± 1.67	16.2 ± 1.44 *	0.01
MIP-1β	46.4 ± 5.71	35.5 ± 2.62	0.37
TARC	49.4 ± 6.28	46.8 ± 4.45	0.39
GM-CSF	0.2 ± 0.15	0.08 ± 0.02	0.41
IL-12p40	113 ± 10.14	116 ± 14.7	0.71
IL-15	2.17 ± 0.09	2.40 ± 0.27	0.74
IL-16	298 ± 30.5	284 ± 26.2	0.91
IL-17A	2.2 ± 0.37	1.51 ± 0.18	0.05
IL-1α	0.24 ± 0.07	0.36 ± 0.10 *	0.03
IL-5	0.32 ± 0.09	0.41 ± 0.17	0.42
IL-7	4.29 ± 0.29	4.11 ± 0.28	0.93
TNF-β	0.30 ± 0.02	0.37± 0.03	0.06
VEGF-A	44.7 ± 3.75	36.5 ± 3.09	0.28

* *p* < 0.05.

**Table 3:** Selected plasma cytokines level in all studied groups (pg/mL).

Cytokines	Unexposed Workers	Radiation Workers			
	Control (*n* = 40)	All (*n* = 20)	Conventional Radiography (CR)	Interventional Radiography (IR)	Computed Tomography (CT)
IL-6					
Range	0.05–2.3	0.17–1.70	0.17–1.70	0.42–1.42	0.3–1.04
Mean ± SEM	0.44 ± 0.08	0.60 ± 0.08 ^a^	0.50 ± 0.12	0.83 ± 0.21 ^b^	0.67 ± 0.15
MIP-1α					
Range	0–46.70	8.86–31.19	8.86–23.62	16.9–31.19	12.25–19.99
Mean ± SEM	12.0 ± 1.67	16.2 ± 1.44 ^a^	13.82 ± 1.56	23.84 ± 3.38 ^b^	15.80 ± 1.62
IL-1α					
Range	0–1.77	0.03–1.79	0.03–1.79	0.03–0.48	0.07–0.63
Mean ± SEM	0.24 ± 0.07	0.36 ± 0.10 ^a^	0.40 ± 0.16	0.26 ± 0.10	0.36 ± 0.14

^a^*p* < 0.05 versus control; ^b^ *p* < 0.05 versus control and CR.

**Table 4:** Plasma 8-OHdG concentration in all studied groups (ng/mL).

Plasma 8-OHdG	Unexposed Workers	Radiation Workers			
	Control (*n* = 40)	All (*n* = 20)	Conventional Radiography (CR)	Interventional Radiography (IR)	Computed Tomography (CT)
8-OHdG concentration					
Range	0.92–8.11	2.22–7.63	2.22–7.63	2.86–5.08	2.89–5.04
Mean ± SEM	4.51 ± 0.40	3.97 ± 0.27	3.92 ± 0.39	4.16 ± 0.49	3.93 ± 0.57

*p* > 0.05.

## Abbreviations

O_2_•^−^	superoxide
EcSOD	extracellular superoxide dismutase
GSH/GSSG	reduced/oxidized glutathione ratio
ICRP	International Commission of Radiation Protection
ROS	reactive oxygen species
mSV	millisieverts
EPR	electron paramagnetic resonance
a.u.	arbitrary units
CR	conventional radiography
CT	computed tomography
IR	interventional radiography
8-OHdG	8-hydroxydeoxyguanosine
IL	interleukin
MIP	macrophage inflammatory protein
